# Permanent draft genome sequence of *Comamonas testosteroni* KF-1

**DOI:** 10.4056/sigs.3847890

**Published:** 2013-05-30

**Authors:** Michael Weiss, Anna I. Kesberg, Kurt M. LaButti, Sam Pitluck, David Bruce, Loren Hauser, Alex Copeland, Tanja Woyke, Stephen Lowry, Susan Lucas, Miriam Land, Lynne Goodwin, Staffan Kjelleberg, Alasdair M. Cook, Matthias Buhmann, Torsten Thomas, David Schleheck

**Affiliations:** 1 Department of Biological Sciences, University of Konstanz, Germany; 2 Konstanz Research School Chemical Biology, University of Konstanz, Germany; 3 DOE Joint Genome Institute, Walnut Creek, California, USA; 4 Los Alamos National Laboratory, Bioscience Division, Los Alamos, New Mexico, USA; 5 Oak Ridge National Laboratory, Oak Ridge, Tennessee, USA; 6 Centre for Marine Bio-Innovation and School of Biotechnology and Biomolecular Science, University of New South Wales, Sydney, Australia

**Keywords:** *Comamonas testosteroni* KF-1, aerobic, Gram-negative, *Comamonadaceae*, xenobiotic surfactant biodegradation

## Abstract

*Comamonas testosteroni* KF-1 is a model organism for the elucidation of the novel biochemical degradation pathways for xenobiotic 4-sulfophenylcarboxylates (SPC) formed during biodegradation of synthetic 4-sulfophenylalkane surfactants (linear alkylbenzenesulfonates, LAS) by bacterial communities. Here we describe the features of this organism, together with the complete genome sequence and annotation. The 6,026,527 bp long chromosome (one sequencing gap) exhibits an average G+C content of 61.79% and is predicted to encode 5,492 protein-coding genes and 114 RNA genes.

## Introduction

*Comamonas testosteroni* strain KF-1 (DSM14576) was isolated for its ability to degrade xenobiotic sulfophenylcarboxylates (SPC), which are degradation intermediates of the synthetic laundry surfactants linear alkylbenzenesulfonates (LAS) [1]. LAS is in use worldwide (appr. 3 × 10^6^ tons per year [2]) and consists of a complex mixture of linear alkanes (C_10_-C_13_) sub-terminally substituted by 4-sulfophenyl rings (i.e., 38 different compounds) [2]. Commercial LAS is completely biodegradable, as known for more than 50 years [3], e.g., in sewage treatment plants, and its degradation is catalyzed by heterotrophic aerobic bacterial communities in two steps. First, an initial degradation step is catalyzed by bacteria such as *Parvibaculum lavamentivorans* DS-1^T^ [4] through activation and shortening of the alkyl-chains of LAS, and many short-chain degradation intermediates are excreted by these organisms, i.e., approximately 50 different SPCs and related compounds [1,5-8]. Secondly, the ultimate degradation step, i.e., mineralization of all SPCs, is catalyzed by other bacteria in the community, and one representative of these is *Comamonas testosteroni* KF-1. In particular, strain KF-1 was isolated from a laboratory trickling filter that had been used to enrich a bacterial community from sewage sludge that completely degraded commercial LAS and SPCs [1,6]. Strain KF-1 is able to utilize four individual SPCs (both enantiomers), namely *R*/*S*-3-(4-sulfopenyl)butyrate (3-C_4_-SPC), enoyl-3-C_4_-SPC, *R*/*S*-3-(4-sulfopenyl)pentanoate (3-C_5_-SPC), and enoyl-3-C_5_-SPC (see therefore also below), as novel carbon an energy sources for its heterotrophic aerobic growth [1,9,10].

The first *Comamonas testosteroni* (formerly *Pseudomonas testosteroni* [11]) strain, type-strain ATCC 11996, was enriched from soil and isolated in 1952 for its ability to degrade testosterone [12,13]. Since then, the physiology, biochemistry, genetics, and regulation of steroid degradation in this and in other *C. testosteroni* strains have been elucidated in great detail [e.g., 14-21]. Most recently, the genome of *C. testosteroni* ATCC 11996^T^ has been sequenced in order to further improve the understanding of the molecular basis for the degradation of steroids [22].

In the environment, members of the genus *Comamonas* may also be important degraders of aromatic compounds other than steroids, especially of xenobiotic pollutants, since they have frequently been enriched and isolated for their ability to utilize (xenobiotic) aromatic compounds. For example, *Comamonas sp.* strain JS46 is able to grow with 3-nitrobenzoate [23], *Comamonas sp.* strain CNB-1 with 4-chloronitrobenzene [24], *C. testosteroni* T-2 with 4-toluenesulfonate and 4-sulfobenzoate [25], *C. testosteroni* WDL7 with chloroaniline [26], *Comamonas sp.* strain JS765 with nitrobenzene [27], *Comamonas sp.* strain B-9 with lignin-polymer fragments [28], *C. testosteroni* B-356 with biphenyl and 4-chlorobiphenyl [29], *Comamonas sp.* strain KD-7 with dibenzofuran [30], *Comamonas sp.* strain 4BC with naphthalene-2-sulfonate [31], or *C. testosteroni* SPB-2 (as well as strain KF-1) with 4‑sulfophenylcarboxylates [1]. In several *C. testosteroni* strains, the physiology, biochemistry, genetics, and/or regulation of the utilization of aromatic compounds have been elucidated [e.g., 10,23,25,27,29,32-48]. Furthermore, the genome sequence of (plasmid-cured) *C. testosteroni* CNB-2 has been published [24], and the sequence of its plasmid pCNB1 (of *C. testosteroni* CNB-1) [49], in order to further improve the understanding of the molecular basis for the ability of *C. testosteroni* to degrade such a large array of aromatic compounds.

Members of the genus *Comamonas* are able to cope with harsh environmental conditions such as high concentrations of arsenate [50,51], zinc [52], cobalt and nickel [53], or phenol [54], and can exhibit increased resistance to oxidative stress [55] or antibiotics [56]. Another *C. testosteroni* genome sequence, of strain S44, has recently been established in order to improve the understanding of the molecular basis for its resistance to increased concentrations of zinc [52]. Notably, an increased antibiotic resistance (and enhanced insecticide catabolism) as a consequence of induction of the steroid degradation pathway has been shown for *C. testosteroni* ATCC 11996^T^ [56].

Here, we present a summary classification and a set of features for another *C. testosteroni* strain, strain KF-1, which has been genome-sequenced in order to improve the understanding of the molecular basis for its ability to degrade xenobiotic compounds, particularly xenobiotic, chiral 3-C_4_-SPC, and how this novel degradation pathway has been assembled in this organism, together with the description of its draft genome sequence and annotation. The genome sequence and its annotation have been established as part of the Microbial Genomics Program 2006 of the DOE Joint Genome Institute, and are accessible *via* the IMG platform [57].

## Classifications and features

### Morphology and growth conditions

**Figure 1:**
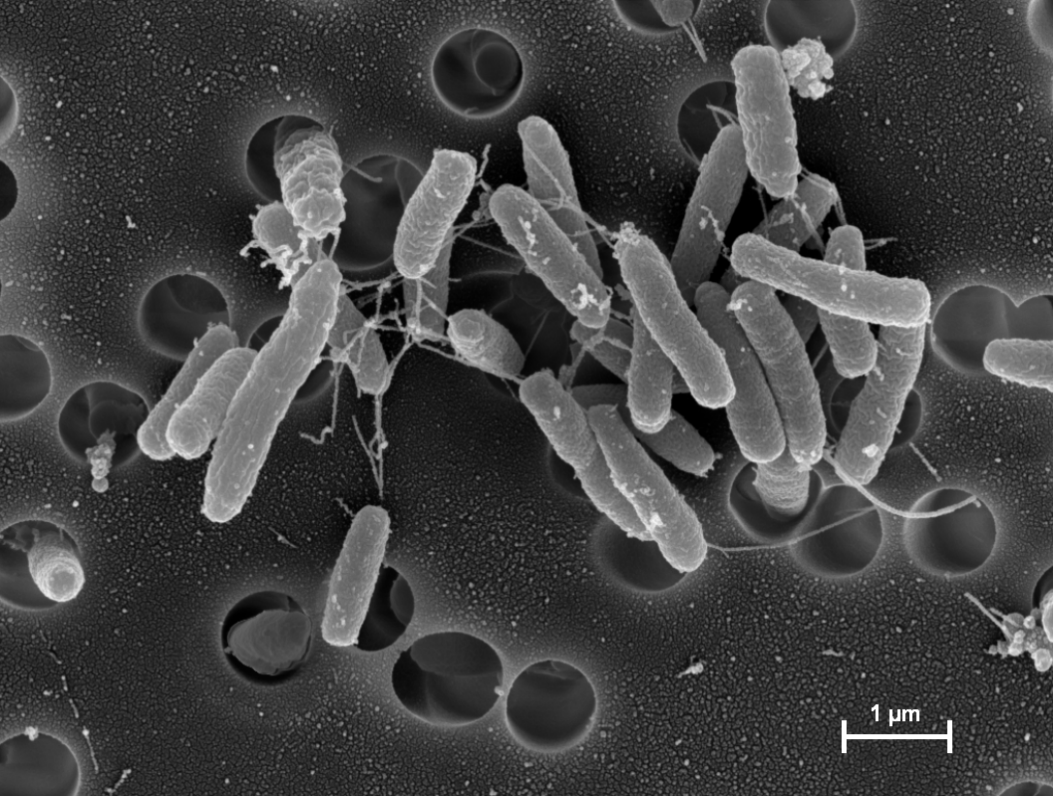
Scanning electron micrograph of *Comamonas testosteroni* KF-1 . Cells derived from a liquid culture that grew in LB medium.

*C. testosteroni* KF-1 is a rod-shaped (size, appr. 0.5 x 2 µm, Figure 1) Gram-negative bacterium that can be motile and grows strictly aerobically with complex medium (e.g., in LB- or peptone medium) or in a prototrophic manner when cultivated in mineral-salts medium [58] with a single carbon source (e.g., acetate). Strain KF-1 grows overnight on LB-agar plates and forms whitish-beige colonies [Table 1]. The strain grew with all amino acids tested (D-alanine, L-alanine, L-aspartate, L-phenylalanine, L-valine, glycine, L-histidine, L-methionine), but not with any of the sugars tested (D-glucose, D-fructose, D-galactose, D-arabinose, and D-maltose). Strain KF-1 utilized the following alcohols and carboxylic acids when tested (in this study): ethanol, acetate, glycerol, glycolate, glyoxylate, butanol, butyrate, isobutyrate, succinate, *meso*-tartaric acid, D- and L-malate, mesaconate, and nicotinate. Furthermore, strain KF-1 was positive for growth with poly-*beta*-hydroxybutyrate (this study). Strain KF-1 is able to utilize the steroids testosterone and progesterone (confirmed in this study), as well as taurocholate and cholate (and taurine and N-methyl taurine) [19], and taurodeoxycholate; strain KF-1 was tested negative for growth with cholesterol, ergosterol, 17β-estradiol and ethinylestradiol (this study), correlating with the findings for *C. testosteroni* strain TA441 [20].

**Table 1:** Classification and general features of *Comamonas testosteroni* KF-1 according to the MIGS recommendations [59].

**MIGS ID**	**Property**	**Term**	**Evidence code**^a^
	Current classification	Domain *Bacteria*	TAS [60]
		Phylum *Proteobacteria*	TAS [61]
		Class *Betaproteobacteria*	TAS [62,63]
		Order *Burkholderiales*	TAS [62,64]
		Family *Comamonadaceae*	TAS [65]
		Genus *Comamonas*	TAS [11,66-69]
		Species *Comamonas testosteroni*	TAS [11,68]
		Strain KF-1	TAS [1]
	Gram stain	Negative	
	Cell shape	small rod	
	Motility	Motile	
	Sporulation	non-sporulating	
	Temperature range	Mesophile	TAS [1]
	Optimum temperature	30ºC	TAS [1]
	Carbon source	3-(4-sulfophenyl)butyrate (3-C_4_-SPC) and other SPCs [see text], 4-sulfoacetophenone, 4-sulfophenyl acetate, 4-sulfophenol, testosterone, progesterone, taurocholate, cholate, taurine, benzoate, 4-hydroxybenzoate, vanillate, isovanillate	IDA,TAS [1,19]
	Energy source	Chemoorganotroph	TAS [1,6]
	Terminal electron receptor	molecular oxygen	TAS [1,6]
MIGS-6	Habitat	aerobic habitat	TAS [1,6]
MIGS-22	Oxygen requirement	Aerobic	TAS [1,6]
MIGS-15	Biotic relationship	free-living	TAS [1,6]
MIGS-14	Pathogenicity	nonpathogenic, Risk group 1 (classification according to German TRBA)	
MIGS-4	Geographic location	isolated from a LAS surfactant-degrading laboratory trickling filter (University of Konstanz, Germany) that had been inoculated with sludge from a communal sewage treatment plant (Herisau, Switzerland).	TAS [1,6]
MIGS-5	Collection date	1999	TAS [1,6]
MIGS-4.1	Latitude	47° 41' 27.24"	TAS [1,6]
MIGS-4.2	Longitude	9° 11' 16.25"	TAS [1,6]
MIGS-4.4	Altitude	440 m	TAS [1,6]

^a^ Evidence codes - IDA: Inferred from Direct Assay; TAS: Traceable Author Statement; NAS: Non-traceable Author Statement. These evidence codes are from the Gene Ontology project [70]. If the evidence is IDA, then the property was directly observed for a live isolate by one of the authors or an expert mentioned in the acknowledgements.

In respect to other aromatic compounds, strain KF-1 is known to utilize benzoate, 3- and 4-hydroxybenzoate, protocatechuate (3,4-dihydroxybenzoate), gentisate (2,5-dihydroxybenzoate), phthalate, terephthalate, vanillate, isovanillate, veratrate, 2- and 3-hydroxyphenylacetate (tested in this study, and ref. 1). Xenobiotic aromatic substrates for strain KF-1 known are the 4-sulfophenylcarboxylates *R*/*S*-3-(4-sulfophenyl)butyrate (*R*/*S*-3-C_4_-SPC), 3-(4-sulfophenyl)-∆2-enoylbutyrate (enoyl-3-C_4_-SPC), *R*/*S*-3-(4-sulfophenyl)pentanoate (*R*/*S*-3-C_5_-SPC), 3-(4-sulfophenyl)-∆2-enoylpentanoate (enoyl-3-C_5_-SPC), as well as the three xenobiotic metabolites in the 3-C_4_-SPC-pathway, 4-sulfoacetophenone (4-acetylbenzenesulfonate), 4-sulfophenol acetate, and 4-sulfophenol [1,9]. Finally, strain KF-1 did not utilize the following, other carbon sources tested (this study and refs. 1,9): *n*-alkanes (C_6_-C_12_), cycloalkanes (C_8_-C_12_), secondary-4-sulfophenylalkanes (LAS surfactants), secondary alkanesulfonates (SAS surfactants), dodecylsulfate (SDS surfactant), benzene sulfonate, 4-toluenesulfonate, 4-sulfobenzoate, phenylacetate, 3-phenylpropionate, 3- and 4-phenylbutyrate, 4-sulfostyrene, 4-sulfobenzoate, 4-sulfocatechol, cyclohexanone, 4-aminoacetophenone, gallic acid (3,4,5-trihydroxybenzoic acid) and gallotannic acid, pentanesulfonate, isethionate, sulfoacetate, D-tartaric acid, acetamide, *gamma*-aminobutyrate, oxalate, methanol, methylamine, methanesulfonate or formate, and not 2-C_4_-SPC (2-[4-sulfophenyl]butyrate), 4-C_5_-SPC, 4-C_6_-SPC, 5-C_6_-SPC, or any of the C_7_ – C_9_ SPCs generated during commercial LAS surfactant degradation.

*C. testosteroni* KF-1 has been recognized for its poor ability to form structured biofilms on surfaces [71] [see also ref. 72], or micro- or macroscopic cellular aggregates in liquid cultures [73], in direct comparison to ‘good’ biofilm forming organisms such as *Delftia acidovorans* SPH-1 [71], *Pseudomonas aeruginosa* PAO1 [73], or *C. testosteroni* SPB-2 [1].

No significant production of siderophores could be observed for *C. testosteroni* KF-1 when grown in presence of non-inhibitory levels of iron chelator 2,2'-dipyridyl [see 74], in comparison to siderophore-producing *Delftia acidovorans* SPH-1, *Pseudomonas aeruginosa* PAO1, and *Pseudoalteromonas tunicata* D2 [75] (reported in this study, data not shown).

Finally, strain KF-1 is able to grow in the presence of up to 500 µg/ml ampicillin or 600 µg/ml kanamycin in liquid cultures, as tested in this study.

### Phylogeny

**Figure 2:**
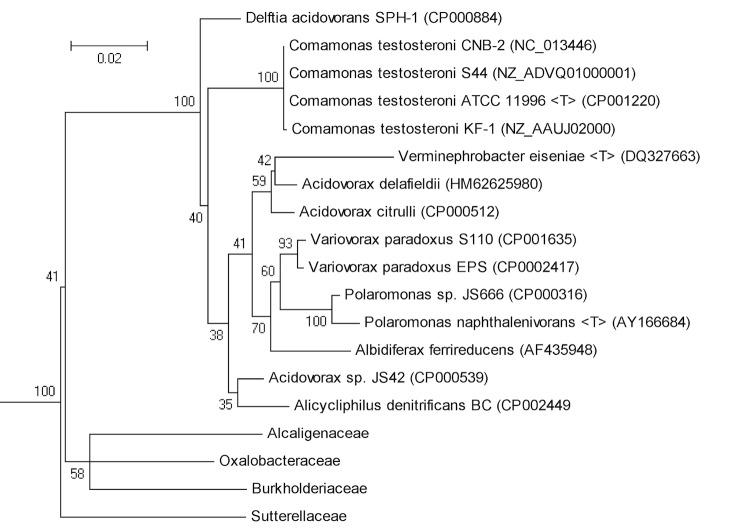
Illustration of the phylogenetic position of *Comamonas testosteroni* KF-1 within the order *Burkholderiales* of *Betaproteobacteria*. The 16S rRNA gene alignment included the three other *C. testosteroni* strains whose genome sequences have been published, strain S44 [52], strain CNB-2 [24], and type-strain ATCC 11996 [22], and some of other genome-sequenced representatives of the family *Comamonadaceae* or of other families within the order *Burkholderiales*. The corresponding genome-project accession numbers, or 16S rRNA gene accession numbers, are indicated. “T” indicates a type strain. The sequences were aligned using the RDP tree builder [76] and displayed using MEGA4 [77]. Bootstrap values are indicated; bar, 0.02 substitutions per nucleotide position.

Based on its 16S rRNA gene sequence, strain KF-1 is a member of the genus *Comamonas*, which is placed in the family *Comamonadaceae* within the order *Burkholderiales* of *Betaproteobacteria*, as illustrated by a phylogenetic tree shown in Figure 2. Currently, 686 genome sequences of members of the order *Burkholderiales* of *Betaproteobacteria*, and 147 genome sequences within the family *Comamonadaceae*, have been, currently are, or are targeted to be established (GOLD database; May 2013).

## Genome sequencing information

### Genome project history

The genome was selected for sequencing as part of the U.S. Department of Energy - Microbial Genomics Program 2006. The DNA sample was submitted in February 2006 and the initial sequencing phase was completed in July 2006. After the finishing and assembly phase the genome was presented for public access on January 2009; a modified version was presented (IMG) in August 2011. Table 2 presents the project information and its association with MIGS version 2.0 compliance [78].

**Table 2:** Project information

**MIGS ID**	**Property**	**Term**
MIGS-31.1	Sequencing status	Complete
MIGS-28	Libraries used	3.5 kb, 9 kb and 37 kb DNA libraries
MIGS-29	Sequencing platforms	Sanger
MIGS-31.2	Sequencing depth	12.8×
MIGS-30	Assemblers	Phred/Phrap/Consed
MIGS-32	Gene calling method	Prodigal
	Genbank ID	17465
	Genbank Date of Release	January 14, 2009
	GOLD ID	Gi01330
MIGS-13	Source material identifier	DSM 14576
	Project relevance	Biotechnological

### Growth conditions and DNA isolation

*Comamonas testosteroni* KF-1, obtained from the Leibniz-Institut DSMZ-Deutsche Sammlung von Mikroorganismen und Zellkulturen (DSM14576), was grown on LB agar plates and transferred into selective medium (6 mM 4-sulfophenol/mineral-salts medium) in the 3-ml scale, and this culture was sub-cultivated in larger scale; cell pellets were stored frozen until DNA preparation. DNA was prepared following the JGI’s DNA Isolation Bacterial CTAB Protocol.

### Genome sequencing and assembly

The genome of *Comamonas testosteroni* KF-1 was sequenced at the Joint Genome Institute (JGI) using a combination of 3.5 kb, 9 kb and 37 kb DNA libraries. All general aspects of library construction and sequencing performed at the JGI can be found at JGI website [79]. In total, 66.91 Mbp of Sanger sequence data were generated for the assembly from all three libraries, which provided for a 12.8-fold coverage of the genome. The Phred/Phrap/Consed software package was used for sequence assembly and quality assessment [80-82]. After the shotgun stage, reads were assembled with parallel phrap (High Performance Software, LLC). Possible mis-assemblies were corrected with Dupfinisher [83], PCR amplification, or transposon bombing of bridging clones (Epicentre Biotechnologies, Madison, WI, USA). Gaps between contigs were closed by editing in Consed, custom primer walk or PCR amplification (Roche Applied Science, Indianapolis, IN, USA). The genome could not be closed due to clone viability issues, however, several clones circularized the contig, and a PCR product was obtained that spanned the ends, but all attempts at primer walking and transforming the amplicon were unsuccessful. At this time no additional work is planned for this project (labeled as Permanent Draft; one linear contig).

### Genome annotation

Genes were identified using Prodigal [84] as part of the genome annotation pipeline at Oak Ridge National Laboratory (ORNL), Oak Ridge, TN, USA, followed by a round of manual curation using the JGI GenePRIMP pipeline [85]. The predicted CDSs were translated and used to search the National Center for Biotechnology Information (NCBI) non-redundant database, UniProt, TIGRFam, Pfam, PRIAM, KEGG, COG, and InterPro databases. Non-coding genes and miscellaneous features were predicted using tRNAscan-SE [86], RNAMMer [87], Rfam [88], TMHMM [89], and signalP [90]. Additional gene prediction analysis and manual functional annotation was performed within the Integrated Microbial Genomes (IMG) platform [91] developed by the Joint Genome Institute, Walnut Creek, CA, USA [92].

## Genome properties

**Figure 3:**
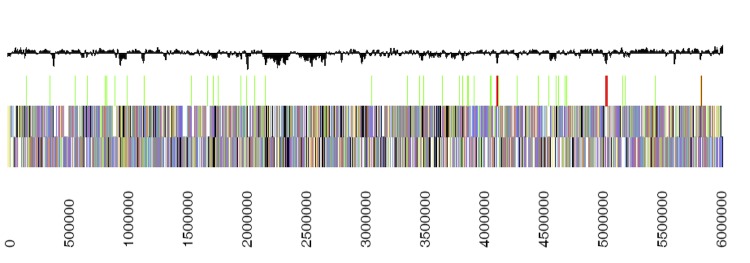
Chromosome map of the genome of *C. testosteroni* KF-1. From bottom to top: Genes on forward strand (colour by COG categories), genes on reverse strand (color by COG categories), RNA genes (tRNA, green; rRNA, red; other RNAs, black), GC content.

The genome of *C. testosteroni* KF-1 comprises a chromosome of 6,026,527 bp (61.76% GC content) (Table 3), for which a total number of 5,606 genes were predicted. Of these predicted genes, 5,492 are protein-coding genes, and 4,009 of the protein-coding genes were assigned to a putative function and the remaining annotated as hypothetical proteins. Genome analysis predicted 114 RNA genes and six rRNA operons. The properties and the statistics of the genome are summarized in Table 3, the distribution of genes into COGs functional categories is presented in Table 4, and the chromosome map of the genome of *C. testosteroni* KF-1 is illustrated in Figure 3.

**Table 3:** Nucleotide and gene count levels of the genome of *C. testosteroni* KF-1

**Attribute**	**Value**	**% of total^a^**
Genome size (bp)	6,026,527	100
DNA coding region (bp)	5,275,818	87.54
DNA G+C content (bp)	3,723,913	61.79
Number of replicons	1	
Extrachromosomal elements	0	
Genes total number	5,606	100
Protein-coding genes	5,492	97.97
RNA genes	114	2.03
rRNA operon count	6	
Genes with function prediction	4,009	71.51
Genes in paralog clusters	1314	23.44
Genes assigned to COGs	4,131	73.69
Genes assigned to Pfam domains	4,375	78.04
Genes connected to KEGG pathways	1,502	26.79
Genes with transmembrane helices	1,265	22.57
Genes with signal peptides	1,410	25.15

^a^) The total is based on either the size of the genome in base pairs or the total number of protein coding genes in the annotated genome.

**Table 4:** Number of genes associated with the general COG functional categories in *C. testosteroni* KF-1

Code	Value	%age	Description
J	187	4.02	Translation, ribosomal structure and biogenesis
A	2	0.04	RNA processing and modification
K	407	8.75	Transcription
L	212	4.56	Replication, recombination and repair
B	2	0.04	Chromatin structure and dynamics
D	32	0.69	Cell cycle control, cell division, chromosome partitioning
Y	-	-	Nuclear structure
V	53	1.14	Defense mechanisms
T	263	5.65	Signal transduction mechanisms
M	239	5.14	Cell wall/membrane/envelope biogenesis
N	102	2.19	Cell motility
Z	-	-	Cytoskeleton
W			Extracellular structures
U	159	3.42	Intracellular trafficking, secretion, and vesicular transport
O	154	3.31	Posttranslational modification, protein turnover, chaperones
C	304	6.54	Energy production and conversion
G	170	3.66	Carbohydrate transport and metabolism
E	361	7.76	Amino acid transport and metabolism
F	90	1.94	Nucleotide transport and metabolism
H	164	3.53	Coenzyme transport and metabolism
I	283	6.08	Lipid transport and metabolism
P	303	6.51	Inorganic ion transport and metabolism
Q	154	3.31	Secondary metabolites biosynthesis, transport and catabolism
R	546	11.74	General function prediction only
S	464	9.98	Function unknown
NA	1475	26.31	Not in COGs

^a^) The total is based on the total number of protein coding genes in the annotated genome.

The chromosome of *C. testosteroni* KF-1 (6.03 Mb) is larger in comparison to these of the three other *C. testosteroni* strains whose sequences have been published, of strain S44 [52] (5.53 Mb), strain CNB-2 [24] (5.46 Mb), and strain ATCC 11996 [22] (5.41 Mb), and in comparison to that of *C. testosteroni* NBRC 100989 (5.59 Mb) whose draft sequence has not yet been published (BioProject ID PRJNA70139). Upon genomic BLAST comparison however, the strain NBRC 100989 chromosome showed the highest similarity to the chromosome of *C. testosteroni* KF-1.

For the three *C. testosteroni* genomes accessible within the IMG platform for direct comparison [57], strains KF-1, S44 and CNB-2, the gene abundance profile indicated, most strikingly, a much higher abundance of transposases (COG2801, COG2826 and COG4644) in strain KF-1 (42 total) in comparison to strains S44 (4 total) and CNB-2 (9 total); retroviral integrases (pfam00665) are more abundant in strain KF-1 (36 total) in comparison to strains S44 (none) and CNB-2 (13 total), and hemagluttinin repeat proteins (pfam05594) implicated in cell aggregation are more abundant (10 total) in comparison to strains S44 (none) and CNB-2 (none).

In respect to candidate genes encoding the metabolic features of *C. testosteroni* KF-1 (see above), almost identical (syntenic) gene clusters were found for the main steroid degradation genes characterized in *C. testosteroni* TA441 [16,17,20], including the genes characterized in *C. testosteroni* ATCC 11996 [18,21,52,93-95]; the strain KF-1 genes are up to 98% identical in their amino-acid sequences. Candidate genes for the degradation of the acyl-sidechain of cholate in *Pseudomonas sp.* strain Chol1 [96,97] were also found (thiolase, locus tag CtesDRAFT_PD3654; acyl-CoA dehydrogenase, PD3666), and the genes for inversion of the cholate-stereochemistry in *Comamonas testosteroni* TA441 [98] (PD3740-44). In respect to the complete degradation of taurocholate [19], several candidate genes for bile-salts hydrolase (taurocholate hydrolase) and candidate genes for the complete degradation of the taurine-moiety (2-aminoethanesulfonate) [19], e.g., for sulfoacetaldehyde acetyltransferase (Xsc, PD0776), were found.

Strain KF-1 has acquired the ability to utilize xenobiotic 3-C_4_-SPC, 3-C_4_-SPC-2H, 3-C_5_-SPC and 3-C_5_-SPC-2H, 4-sulfoacetophenone (SAP), and 4-sulfophenol (SP) (see above) [1,9]. The 3-C_4_-SPC is converted to SAP [9] and further to 4-sulfophenol acetate (SPAc) by a recently identified Baeyer-Villiger monooxygenase (‘SAPMO’, PD5437), and SPAc hydrolyzed by a recently identified carboxylester hydrolase encoded by the next gene in the genome (PD5438), to yield acetate and SP [10]. The two identified genes, together with other (predicted) catabolic genes, are framed by IS*1071* insertion sequence elements (Tn*3*-family transposase genes), which suggests that these genes have only recently been acquired, possibly in the form of a ‘catabolic composite transposon’ through horizontal gene transfer [10]. Genes for other sections of the proposed 3-C_4_-SPC degradation pathway in strain KF-1, i.e., the ‘upper’ and ‘lower’ pathway, from 3-C_4_-SPC to SAP and from SP further to central metabolites, respectively [9], are examined in our present work (unpublished).

*C. testosteroni* KF-1 encodes a wealth of genes for aromatic ring-cleavage oxygenases and aromatic-ring hydroxylating oxygenase (systems), as commonly observed for members of the order *Burkholderiales* [99]. Firstly, the complete protocatechuate 4,5-cleavage *(meta)* degradation operon (*pmd*-operon) characterized in *C. testosteroni* strain BR6020 [35,43], strain E6 [47] and CNB-1 [48] involved in the degradation pathways for vanillate, isovanillate and 3- and 4-hydroxybenzoate, was found in strain KF-1 (*pmdB*, PD1898) (and two *pmdB* paralogs, PD1614 and 1810). An ortholog of the 3-hydroxybenzoate monooxygenase characterized in *C. testosteroni* GZ39 [100] was found in strain KF-1 (PD1242), as were the genes for conversion of vanillate and isovanillate (*vanA*/*ivaA*: PD0400/PD0403) [43].

Gene clusters of the *meta*-pathway enzymes for degradation of phenol as characterized in *C. testosteroni* TA441, i.e., *aphCEFGHJI* [101] and *aphKLMNOPQB* [102]), were not found in strain KF-1, but in strains S44 and CNB-2. However, homologs for all *meta*-pathway enzymes (corresponding to *aphCEFGHJI*) seem to be distributed at different locations in the strain KF-1 genome, but a valid candidate gene cluster of the phenol hydroxylase components (*aph-* [102] or *phcKLMNOP* [34] genes) and catechol 2,3-dioxygenase (*aphB*) could not be found in the strain KF-1 genome. Also the gene cluster for the 3-(3-hydroxyphenyl) propionic acid degradation pathway (*mhp*-operon) characterized in *Comamonas testosteroni* TA441 [103] was not found in the genome of strain KF-1, nor in strain CNB-2, but was found in strain S44; homologs for all pathway enzymes (corresponding to *mhpABDFE)* seem to be distributed at different locations in the strain KF-1 genome.

An almost identical gene cluster for the terepthalate (benzene-1,4-dicarboxylic acid) pathway (*tph*-cluster) as characterized in *C. testosteroni* YZW-D [104] and strain E6 [44,46] was found in strain KF-1 (*tphA*, PD2130). The gene cluster for the isophthalate (benzene-1,3-dicarboxylic acid) pathway of *C. testosteroni* YZW-D [104] and strain E6 [45] was also found in strain KF-1 (*iphA*, PD2139), encoded directly upstream of the *tph*-cluster. Notably, at least nine other Rieske-domain ring-hydroxylating oxygenase component genes (COG4638) similar to *tpaA*/*iphA* (PD2130/PD2139) and *vanA*/*ivaA* (see above, PD0400/PD0403), seem to be encoded in strain KF-1 (PD2042, 1888, 4205, 2022, 0968, 3693, 1612, 2032, 5293).

No ortholog of the catechol 2,3-ring cleavage dioxygenase (non-heme Fe^2+^) of the phenol-pathway gene cluster (*aphB*) [102] was found in strain KF-1, but two other class I/II extradiol ring-cleavage dioxygenase candidates (PD0021, 5290) in addition to a (decarboxylating) 4-hydroxyphenylpyruvate dioxygenase candidate (PD0347) (also in CNB-2 and S44), *tesB* of the steroid gene cluster (PD3739), and the class-III type extradiol ring-cleavage dioxygenases mentioned above (PmdAB) were found.

In respect to intradiol ring-cleavage dioxygenases, three candidates for (non-heme Fe^3+^) catechol 1,2-dioxygenase/protocatechuate 3,4-dioxygenase beta subunit/hydroxyquinol 1,2-dioxygenase were found in strain KF-1, i.e., PD0424, 5469, and 5471; notably, the latter two candidates are not represented in strains CNB-2 and S44.

Also not represented in the *C. testosteroni* KF-1 genome is the nitrobenzene (*nbz*) degradation gene cluster of *Comamonas sp.* JS765 [38], the 3-nitrobenzoate (*mnb*) degradation cluster of *C. testosteroni* BR6020 [23], the 4-chlorobenzoate uptake and degradation cluster of *Comamonas sp.* strain DJ-12 [51,105], and not the 4-chloronitrobenzene (*cnb*) cluster on plasmid pCNB1 in *C. testosteroni* CNB-1 [49] and the upper-pathway chloroaniline (*dca*) cluster on plasmid pWDL7 in *C. testosteroni* WDL7 [26]. Finally, an ortholog of the aliphatic nitrilase/cyanide hydratase (NitA) characterized in a *C. testosteroni* soil isolate [106] was also not found in the genome of strain KF-1, nor in those of CNB-2 or S44.

Strain KF-1 utilized none of the sugars tested (see above), and this observation is reflected by an absence of appropriate candidate genes in strain KF-1 for hexokinase and glucokinase in glycolysis, as well as of genes of the oxidative branch of the pentose phosphate pathway, as reported also for *C. testosteroni* CNB-2 [24].

Strain KF-1 is able to utilize nicotinate for growth and encodes an orthologous set of genes for the nicotinate dehydrogenase /hydroxylase complex (PD0815-13) characterized in *C. testosteroni* JA1 [107].

The poly(3-hydroxybutyrate) (PHB) biosynthesis and utilization operon of *Comamonas sp.* EB172 [108] is also encoded in strain KF-1 (e.g., PD2272). Furthermore, strain KF-1 tested positive for growth with extracellular poly(3-hydroxybutyrate) (this study), and strain KF-1 encodes an ortholog (PD3795) of the characterized poly(3-hydroxybutyrate) depolymerase precursor (PhaZ) of *Comamonas sp.* strain 31A [109]; notably, the ortholog was also found in *C. testosteroni* ATCC 11996^T^, but not in strains S44 and CNB-2.

In respect to the ampicillin (*beta*-lactam) antibiotic resistance of strain KF-1, the genome encodes at least two *beta*-lactamase class A (PD2722, 4357) and one *beta*-lactamase class B (PD0340) candidates, and with respect to kanamycin (aminoglycoside) resistance, two aminoglycoside phosphotransferase candidates (PD3717, 1418); notably, the latter two are not represented in strains CNB-2 and S44.

All four heavy metal exporter ATPase genes (*zntA*) and five CzcA-family exporter gene clusters described for highly zinc-resistant *C. testosteroni* S44 [52] were found in strain KF‑1, and in total eight *zntA* and 11 *cntA* candidates. Two arsenical resistance gene clusters (PD1708-06 and 3544-42), each with candidates for arsenical pump (ArsB), arsenate reductase (ArsC), NADPH:FMN oxidoreductases (ArsH), and transcriptional regulator (ArsR), and a third *arsC* candidate (PD0567), were found in strain KF-1.
